# Progressive Myopenia and Functional Decline in the Winnie Mouse Model of Chronic Colitis

**DOI:** 10.3390/muscles5020038

**Published:** 2026-05-12

**Authors:** Shilpa Sharma, Danielle Debruin, Jeannie Devereaux, Alan Hayes, Kulmira Nurgali, Gustavo Duque

**Affiliations:** 1 Department of Medicine—Western Health, Australian Institute for Musculoskeletal Science (AIMSS), The University of Melbourne, Melbourne, VIC 3021, Australia; 2 Institute for Health and Sport, Victoria University, Melbourne, VIC 3011, Australia; 3 Division of Geriatric Medicine, Department of Medicine, McGill University, Montreal, QC H4A 3J1, Canada

**Keywords:** myopenia, chronic colitis, *Winnie* mouse, muscle atrophy, LCN-2

## Abstract

Muscle wasting contributes substantially to inflammatory bowel disease (IBD)-related disability, but its association with colitis severity across disease stages remains poorly characterized. We therefore assessed skeletal muscle mass, fiber morphology, and voluntary wheel-running performance in *Winnie* mice—a spontaneous *Muc2* mutant model of chronic colitis—in separate female and male homozygous mutant and WT littermate cohorts. Assessments were performed at 5 weeks, before overt colitis, and at 15 weeks, in a cohort with more pronounced colitis. Outcomes included disease activity index (DAI), fecal lipocalin-2 (LCN-2), wheel-running metrics, soleus and tibialis anterior mass, and minimal Feret’s diameter distributions. At 5 weeks, *Winnie* mice showed no overt disease activity and no consistent structural muscle deficit. In contrast, the 15-week cohort exhibited marked colitis in both sexes, with increased DAI and LCN-2, reduced voluntary wheel-running performance, lower soleus and tibialis anterior mass, and smaller muscle fiber diameters with left-shifted size distributions. Correlation analyses identified associations between fecal LCN-2, skeletal muscle mass and size, and wheel-running distance and velocity, supporting a link between intestinal inflammation and muscle impairment in this model. These cross-sectional data are consistent with reduced voluntary activity and structural myopathy during progression of spontaneous colitis. The *Winnie* mouse model therefore provides a clinically relevant preclinical platform to study IBD-associated muscle wasting and its association with intestinal inflammation.

## 1. Introduction

Inflammatory bowel disease (IBD) is a chronic inflammatory disorder primarily targeting the gastrointestinal tract [1]. IBD encompasses ulcerative colitis (UC), characterized by mucosal and submucosal ulcerations confined to the rectum and colon, and Crohn’s disease (CD), which involves transmural inflammation with skip lesions throughout the gastrointestinal tract [2]. Beyond gut pathology, IBD causes systemic complications including myopenia, pre-sarcopenia, and sarcopenia [3]. Progressive myopenia/sarcopenia increases morbidity through falls, fractures, functional decline, hospitalizations, and reduced quality of life [4,5]. Sarcopenia refers to age-related muscle loss with standardized criteria, whereas myopenia refers to disease-induced muscle wasting [6]. Its multifactorial etiology includes chronic inflammation, physical inactivity, malnutrition, and corticosteroid exposure [7,8]. However, the temporal relationship between intestinal inflammation and skeletal muscle deterioration remains poorly defined.

Previous studies have demonstrated muscle wasting in acute chemically induced colitis models, such as 2,4,6-trinitrobenzene sulfonic acid (TNBS) or dextran sulfate sodium (DSS) as well as in IL-10^−/−^ mice [9,10,11,12]. However, these models have important limitations, including short disease duration, epithelial injury that does not fully recapitulate chronic IBD pathology, and microbiome- or sex-dependent confounding [13,14,15,16,17].

The *Winnie* mouse addresses this gap because the *Muc2* mutation causes spontaneous colitis that is detectable from an early pre-clinical stage at around 5 weeks and is more advanced by 15 weeks. This model recapitulates UC-like pathology through goblet cell dysfunction, a reduced mucosal barrier integrity, and downstream IL-23/Th17-driven inflammation [18,19,20,21,22,23,24].

We hypothesized that chronic colitis in *Winnie* mice would be associated with differences in voluntary physical activity and skeletal muscle properties across age-stratified cohorts, with more pronounced functional and structural abnormalities in mice sampled at 15 weeks compared with those sampled at 5 weeks. We further hypothesized that these age-stratified changes would correspond to intestinal inflammation severity. Age- and sex-matched homozygous littermate controls enabled assessment of in vivo wheel running and ex vivo tibialis anterior (TA) and soleus (SOL) muscle properties.

## 2. Methods

### 2.1. Study Design and Experimental Animals

We conducted an experiment using age- and sex-matched homozygous *Winnie*^−/−^ (*Muc2* mutant) mice and *Winnie*^+/+^ littermate controls. *Winnie*^−/−^ (*Muc2*^Cys271Tyr^ homozygous mutant; designated “*Winnie*”) and age- and sex-matched *Winnie*^+/+^ littermate controls (designated “WT”) were bred from heterozygous *Winnie*^+/−^ parents at the Victoria University Werribee Animal Facility (Melbourne, VIC, Australia). Littermates were randomly allocated to experimental groups at weaning. Animals were housed under identical conditions, including cage location, chow diet, temperature (23 ± 2 °C), humidity (55 ± 5%), and a 12 h light/dark cycle, to minimize confounding.

Separate cohorts were assessed at 5 weeks (pre-colitis) and 15 weeks (established colitis). A total of 64 mice were enrolled for the initial Promethion wheel-running assessment, with 8 mice per sex × genotype group at each age (Table 1). The same cohort of mice was reused for subsequent assays where feasible, with endpoint-specific subset sizes determined by assay feasibility and tissue allocation. Central nucleation was quantified only in 15-week mice, when overt structural myopenia was present. Additional secondary analyses included correlation analyses between fecal LCN-2 and muscle outcomes in 15-week mice. No a priori exclusion criteria were applied, and all animals remained in the study cohort. Sample size was estimated using G*Power v3.1.9.7 (power = 0.82, α = 0.05) to detect effect sizes of at least 25% based on pilot data. All procedures complied with the Australian Code and were approved by the Victoria University Animal Ethics Committee (17/016).

### 2.2. Colitis Severity Assessment

Disease parameters in *Winnie* mice were scored in comparison to WT mice based on the percentage of weight loss (e.g., 0 = no weight loss, 1 = 1–5% weight loss, 2 = 5–10% weight loss, 3 = 10–20% weight loss, 4 = >20% weight loss), stool consistency (0 = normal, 1 = loose stools, 2 = diarrhea), and rectal bleeding (0 = no blood, 1 = trace, 2 = gross). The DAI is calculated as the sum of the scores for weight loss, stool consistency, and rectal bleeding.

### 2.3. Fecal Lipocalin-2 Measurement

To assess colonic inflammation levels, fecal lipocalin-2 (LCN-2) was quantified using a commercial ELISA kit (ab119601, Mouse lipocalin-2/NGAL ELISA kit, Abcam, Cambridge, UK) [25]. Fresh fecal pellets were collected, snap-frozen in liquid nitrogen, and stored at −80 °C. Frozen fecal samples were homogenized in phosphate-buffered saline (PBS) containing 0.1% Tween-20 (100 mg/mL, 50 μL/sample) and vortexed for 20 min. Following centrifugation (14,000 rpm, 10 min, 4 °C), clear supernatants were collected. Serial dilutions of the standard Lcn-2 were prepared according to manufacturer’s instructions, generating a standard curve (3.3–3333 pg/mL). 50 μL samples/standards were added to appropriate wells, followed by 50 μL antibody cocktail (prepared by diluting 10× capture and detector antibodies in antibody diluent). Plates were sealed and incubated for 1 h at room temperature with shaking (400 rpm). Wells were washed with wash buffer (3 × 350 μL), followed by addition of TMB development solution (100 μL) and incubation for 10 min in the dark with shaking. The reaction was stopped with stop solution (100 μL), and OD was measured at 450 nm.

### 2.4. Physical Activity Analysis

Mice were individually housed in Promethion metabolic cages (Sable Systems International, North Las Vegas, NV, USA) at temperature 23 ± 2 °C, humidity 55 ± 5%, 12 h light/dark cycle, ad libitum standard chow and water with running wheel access. Following 4-day acclimatization, final 24 h data (diurnal 0700–1900 h; nocturnal 1900–0700 h) were analyzed in 4 min bins via ExpeData software macro 6 (version 1.9.27). Endpoints measured were wheel distance/velocity/time, X-ambulation, Z-rearing and % inactivity.

### 2.5. Muscle Histomorphometry

Post-euthanasia, SOL and TA muscles were immediately excised, weighed, and embedded in an optimal cutting temperature (OCT) compound. The muscles were then snap-frozen in partially thawed isopentane (Sigma Aldrich, Castle Hill, NSW, Australia) and stored at −80 °C until histological analysis. Transverse muscle sections (12 µm thick) were cut from the mid-belly region of each muscle in a cryotome at −15 °C and then air-dried for 30 min. For hematoxylin and eosin (H&E) staining, air-dried cryosections were immersed in hematoxylin (Sigma-Aldrich) for 1 min, followed by a tap water rinse (30 s). Sections were then immersed in Scott’s tap water for 1 min for bluing, rinsed again in tap water (30 s), and counterstained in eosin (Sigma-Aldrich) for 3 min. Following a final tap water rinse (30 s), sections were dehydrated through ascending ethanol gradients (70%, 95%, 100%; 2 min each), cleared in xylene (Sigma-Aldrich) for 5 min, and mounted using DPX mounting medium (BDH, Poole, UK).

Muscle cross-sections were visualized using a Zeiss Axio Imager Z2 microscope (Carl Zeiss Microimaging, Jena, Germany) at 20× magnification. Images were acquired using the Metasystems Metaphor program (Version 4.3). Subsequently, these images were digitally stitched together to create composite views of the entire muscle cross-section using VSlide software (Version 4.3). Muscle fiber size was quantified using the minimal Feret’s diameter method. Minimal Feret’s diameter represents the minimum distance between two parallel tangents at opposing borders of the muscle fiber, regardless of orientation. This method was chosen for its robustness against variations in tissue sectioning angle, which can significantly affect other size parameters [26]. 150–200 fibers/muscle were analyzed for each animal. Minimal Feret’s diameters were measured and recorded using ImageJ software (https://imagej.net) (National Institutes of Health, Bethesda, MD, USA), with measurements calibrated against a stage micrometer for accuracy. The percentage of fibers containing centrally located nuclei was quantified from 200 fibers as an indicator of muscle regeneration. During allocation, investigators were aware (genotype determination required). During experiment: not blinded (colitis monitoring required genotype knowledge). All histological analyses blinded to genotype.

### 2.6. Muscle Selection

Hindlimb muscles TA and SOL were examined in *Winnie* vs. WT mice, as lower extremity mass loss predominates in sarcopenia [27]. Mouse soleus expresses closest molecular resemblance to human skeletal muscles [28] and generates force/work via fascicle-tendon interaction during walking/running [29]. This selection elucidates phenotypic effects of spontaneous chronic colitis on skeletal muscle.

### 2.7. Statistical Analysis

Data are presented as mean ± standard error of mean (SEM). Normality was assessed using Shapiro–Wilk test. Outcomes were analyzed using factorial ANOVA models with genotype, sex, and age/time as fixed factors, with interaction terms examined in the full model. Where significant interactions were present, simple-effects comparisons or sex-stratified follow-up analyses were performed as appropriate, with Tukey’s multiple-comparisons test used for post hoc pairwise comparisons. Promethion wheel-running outcomes were analyzed using a mixed-effects repeated-measures model, with day and night treated as repeated measures within mouse and genotype and age/time as fixed factors. DAI was analyzed as a bounded score with a floor at 0; WT mice had DAI values of 0 at both ages, reflecting absence of clinical disease and producing a floor effect rather than missing data. These zero values were retained as valid observations and included in the prespecified analysis. Spearman correlations were used to assess non-parametric relationships. Male and female data are shown separately for clarity, but statistical inference was based on the full factorial model including genotype, sex, age/time, and interaction terms. *p* < 0.05 was considered statistically significant.

## 3. Results

### 3.1. Progressive Colitis in Winnie Mice

**Figure 1:**
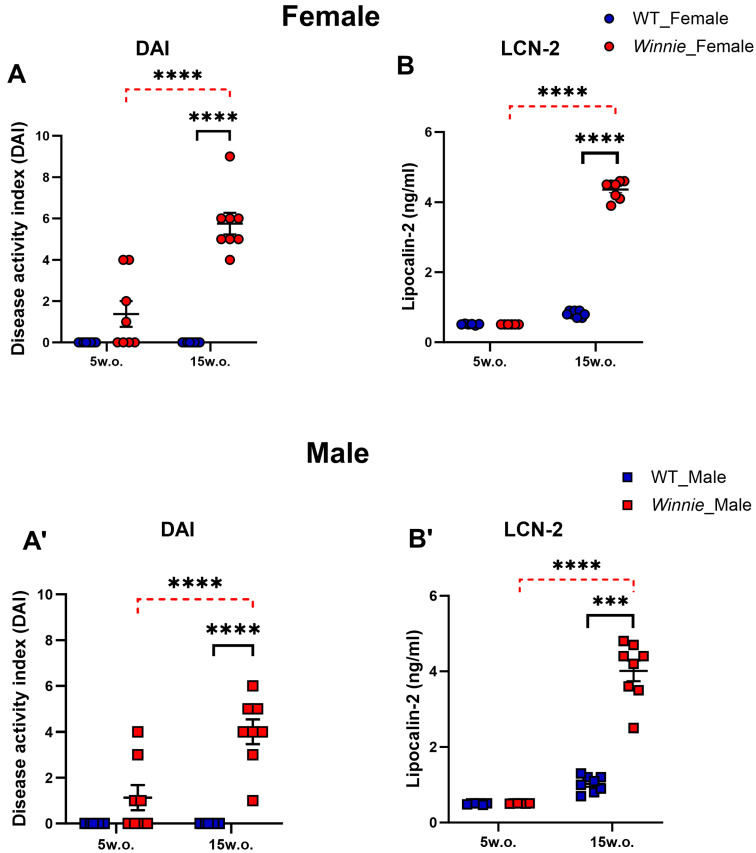
Colitis progression in *Winnie* mice. Disease Activity Index (DAI) of female and male *Winnie* vs. WT mice at 5 and 15 weeks (**A**,**A′**). Fecal lipocalin-2 (LCN-2) levels of female and male *Winnie* vs. WT mice at 5 and 15 weeks (**B**,**B′**). Data are mean ± SEM. Each symbol represents one mouse. Dashed red lines indicate significant age-related comparisons within *Winnie* mice, and solid black lines indicate significant genotype comparisons within each age group. *n* = 8 mice/group for DAI and *n* = 6 mice/group for 5 weeks and 8 mice/group for 15 weeks for the lipocalin assay. *** *p* < 0.001, **** *p* < 0.0001.

At 5 weeks, DAI values were negligible in WT mice and low in *Winnie* mice, consistent with the absence of overt clinical colitis. At 15 weeks, *Winnie* mice exhibited active colitis: DAI significantly elevated (*p* < 0.0001 for females and males both) with loose stools/bleeding, indicating clear progression of disease with age (Figure 1A,A′). Fecal LCN-2 levels, a robust marker of intestinal inflammation, were similar in 5-week-old *Winnie* and age- and sex-matched WT mice. However, LCN-2 levels were elevated in 15-week-old *Winnie* mice compared to age- and sex-matched WT mice (females and males: *p* < 0.000001) (Figure 1B,B′). DAI and LCN-2 showed progressive, genotype-dependent changes with age. Fecal LCN-2 showed a strong age × genotype interaction (*p* < 0.0001), demonstrating that intestinal inflammation progressed in an age-dependent and genotype-dependent manner.

### 3.2. Functional Decline with Disease Progression in Winnie Mice

Real-time voluntary activity was monitored throughout 24 h periods to assess wheel-running behavior. At 5 weeks, when overt colitis was not yet detectable, female *Winnie* mice showed a reduction in daytime wheel time (63% decrease, *p* < 0.01) and a modest increase in resting time (5% increase, *p* < 0.01), indicating an early change in voluntary activity. Also, wheel-running distance showed a significant effect of day-night time (*p* < 0.0001) and genotype (*p* < 0.0001), as well as a significant time × genotype interaction (*p* < 0.0001) at 5 weeks. These findings indicate that the effect of genotype on wheel-running distance was present at 5 weeks and varied across the day-night cycle. Wheel-running distance showed a significant age × genotype interaction (*p* < 0.0001), indicating that the effect of genotype on running distance became more pronounced with age. At 5 weeks, differences between *Winnie* and WT mice were minimal, whereas by 15 weeks *Winnie* mice showed a clear reduction in running distance. preceding structural atrophy. *Winnie* mice exhibited progressive impairment. Also, wheel velocity (*p* < 0.01), wheel time (*p* < 0.0001), *X*-axis activity (*p* < 0.0001), and *Z*-axis activity *p* < 0.0001) showed a significant effect of time, while genotype and sex were not significant, indicating that early activity changes were subtle and largely circadian in nature.

**Figure 2:**
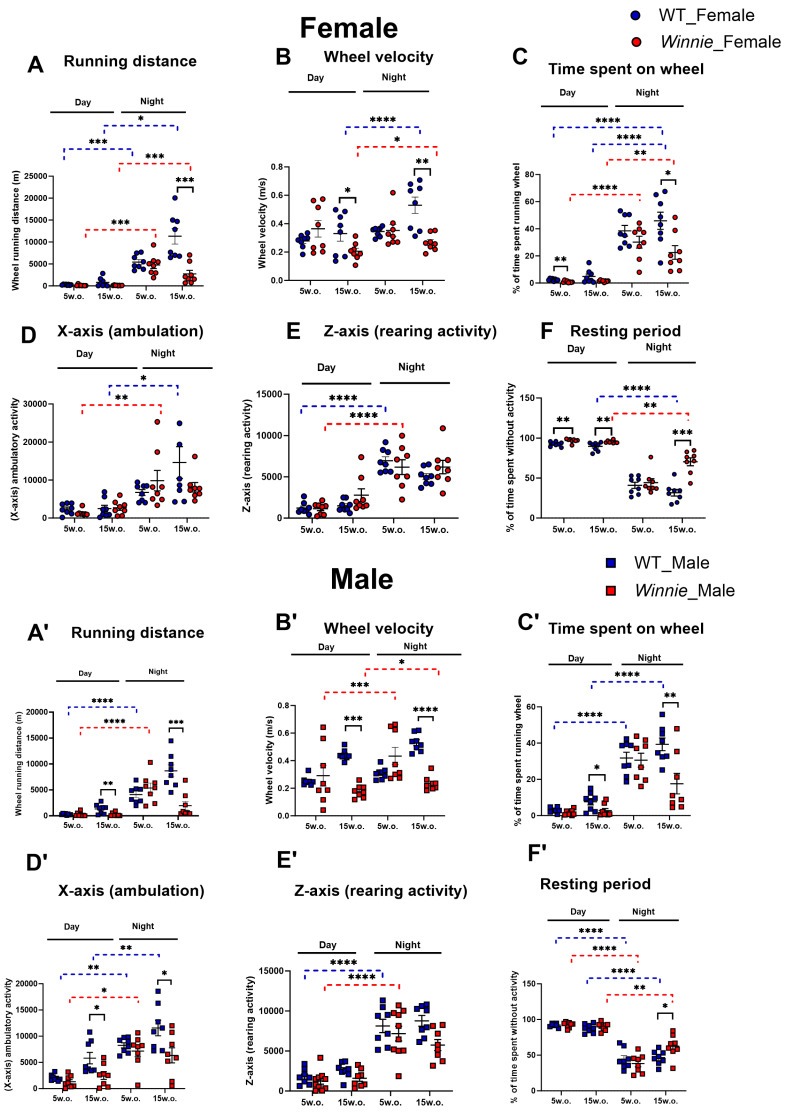
Impaired physical activity of *Winnie* mouse model of colitis at 5 and 15 weeks. Wheel distance (**A**,**A′**), Wheel velocity (**B**,**B′**), Wheel time (%) (**C**,**C′**), X-ambulation (**D**,**D′**), Z-rearing (**E**,**E′**), Rest (%) (**F**,**F′**). Data are mean ± SEM. Each symbol represents one mouse. Day and night phases are shown separately above each panel. Dashed blue lines indicate significant day-versus-night comparisons within WT mice, dashed red lines indicate significant day-versus-night comparisons within *Winnie* mice, and solid black lines indicate significant genotype comparisons within the same age and phase. *n* = 8 mice/group. * *p* < 0.05, ** *p* < 0.01, *** *p* < 0.001, **** *p* < 0.0001.

At 15 weeks, the wheel-running phenotype was markedly more pronounced. Female *Winnie* mice showed reduced nocturnal running distance (−75%, *p* < 0.001). Males showed more deficits in running distance: during daytime (−82%, *p* < 0.01) and nocturnal distance (−77%, *p* < 0.001) (Figure 2A,A′). Wheel-running distance showed a significant age × genotype interaction (*p* < 0.0001), indicating that the effect of genotype on running distance became more pronounced with age. At 5 weeks, differences between *Winnie* and WT mice were minimal, whereas by 15 weeks *Winnie* mice showed a clear reduction in running distance. Wheel-running distance showed significant effects of time (*p* < 0.0001), genotype (*p* < 0.001), and sex (*p* < 0.0001), together with significant time × genotype (*p* < 0.001), time × sex (*p* < 0.0001), genotype × sex (*p* < 0.001), and time × genotype × sex (*p* < 0.001) interactions. These findings indicate that the effect of genotype on running distance varied by sex and across the day-night cycle. Female *Winnie* mice showed decreased wheel velocity during daytime (−37%, *p* < 0.05) and nighttime (−49%, *p* < 0.01) at 15 weeks. Males showed deficits in wheel velocity: during the day (−59%, *p* < 0.001) and night (−55%, *p* < 0.0001) (Figure 2B,B′). Wheel velocity showed a significant age × genotype interaction (*p* < 0.0001), indicating that the genotype effect on velocity increased with age. At 5 weeks, wheel velocity was relatively similar between groups, but by 15 weeks *Winnie* mice showed a marked decline in velocity. Wheel velocity was also significantly affected by time (*p* < 0.0001) and genotype (*p* < 0.0001), with significant time × genotype (*p* < 0.0001), time × sex (*p* < 0.01), and time × genotype × sex effects (*p* < 0.05), while sex and genotype × sex were not significant. These results indicate that velocity deficits were strongly modified by circadian phase and, to a lesser extent, by sex. Female *Winnie* mice exhibited (−50%, *p* < 0.05) reduced nocturnal wheel time at 15 weeks. Males showed similar reductions (−64%, *p* < 0.05) during daytime and night time (−55%, *p* < 0.01) (Figure 2C,C′). Wheel time showed a significant age × genotype interaction (*p* < 0.0001), indicating that the effect of genotype on time spent on the wheel changed with age. *Winnie* mice showed little difference from WT mice at 5 weeks, whereas a clear reduction in wheel time was evident at 15 weeks. Wheel time showed significant effects of time (*p* < 0.0001) and genotype (*p* < 0.0001), but no significant sex effect or sex-related interactions, indicating that the genotype effect on time spent on the wheel varied across the day-night cycle but was not detectably modified by sex.

Unlike females, male *Winnie* mice showed significant reductions in *X*-axis ambulation at 15 weeks during day (−57%, *p* < 0.05) and night (−71%, *p* < 0.05) (Figure 2D,D′). *X*-axis ambulatory activity showed a significant effect of time (*p* < 0.0001), genotype (*p* < 0.01), with a significant time × genotype interaction (*p* < 0.01), while sex and the remaining interaction terms were not significant. This suggests that genotype-associated differences in *X*-axis activity were phase-dependent but not sex-dependent. No significant differences were observed in *Z*-axis rearing activity in both sexes in *Winnie* and WT mice (Figure 2E,E′). *Z*-axis activity showed a significant age × time effect (*p* < 0.01) and a significant genotype × sex interaction (*p* < 0.01). *Winnie* females exhibited increased resting time at 5 weeks (+5%, *p* < 0.01) during daytime and at 15 weeks during daytime (+6%, *p* < 0.001) and nighttime (+122%, *p* < 0.001). *Winnie* males showed increased resting time at 15 weeks (+34%, *p* < 0.05) during night (Figure 2F,F′). Quiet time was significantly affected by time (*p* < 0.0001) and genotype (*p* < 0.0001), with significant time × genotype (*p* < 0.0001), genotype × sex (*p* < 0.05), and time × genotype × sex (*p* < 0.05) interactions, indicating that inactivity also varied by sex and genotype across the day-night cycle. *Winnie* mice become progressively more impaired with age and is shaped by genotype, sex, and circadian phase, with the strongest deficits at 15 weeks.

### 3.3. Muscle Wasting in Winnie Mice at Established Colitis

**Figure 3:**
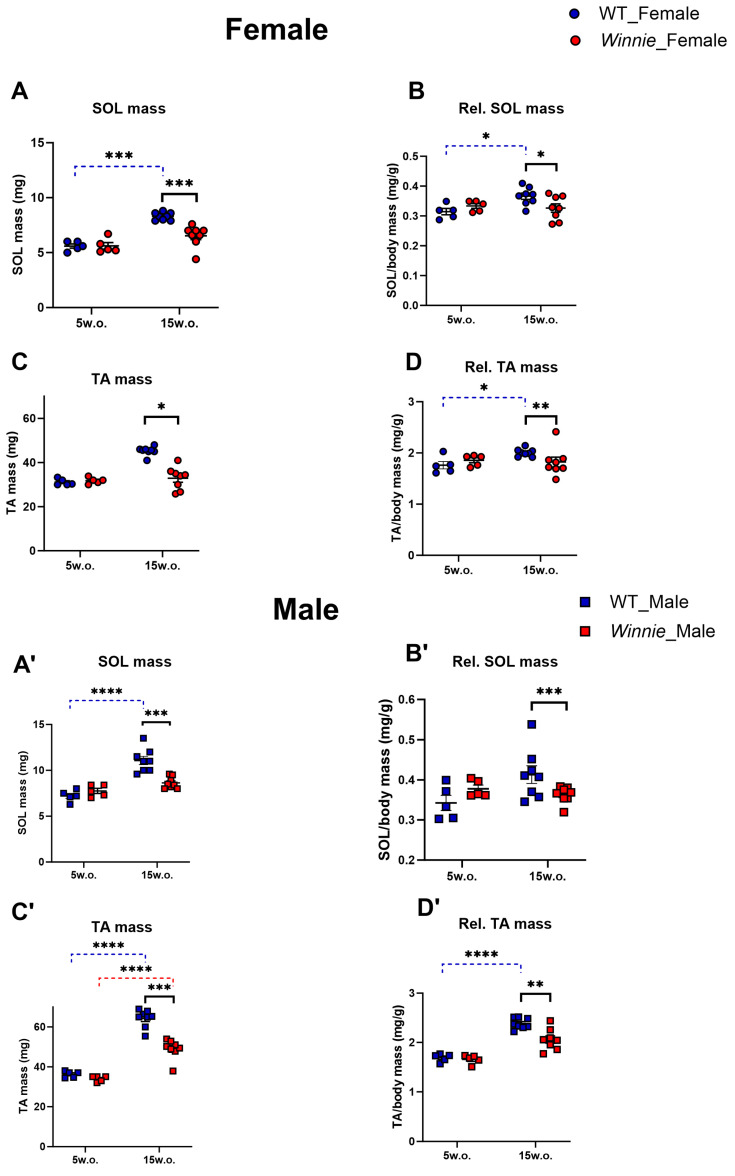
Muscle mass of SOL and TA of *Winnie* mouse model of colitis at 5 and 15 weeks. Absolute SOL mass of female and male *Winnie* mice compared to WT mice at 5 and 15 weeks (**A**,**A′**). SOL mass normalized to body weight of female and male *Winnie* mice compared to WT mice at 5 and 15 weeks (**B**,**B′**). Absolute TA mass of female and male *Winnie* mice compared to WT mice at 5 and 15 weeks (**C**,**C′**). TA muscle normalized to body weight of female and male *Winnie* mice compared to WT mice at 5 and 15 weeks (**D**,**D′**). Data are mean ± SEM. Each symbol represents one mouse. Dashed blue lines indicate significant age-related comparisons within WT mice, dashed red lines indicate significant age-related comparisons within *Winnie* mice, and solid black lines indicate significant genotype comparisons within the same age group. *n* = 5 mice/group at 5 weeks and *n* = 8 mice/group at 15 weeks for muscle mass analyses., * *p* < 0.05, ** *p* < 0.01, *** *p* < 0.001, **** *p* < 0.0001.

To understand the structural basis for the observed functional impairments, we examined muscle mass. At 5 weeks of age, no significant differences were observed in muscle mass between *Winnie* and WT mice for both SOL and TA muscles. At 15 weeks, *Winnie* mice exhibited significant muscle wasting across both sexes and muscle types. Absolute SOL muscle mass was decreased (−21% for females, *p* < 0.001; −22% for males, *p* < 0.001) (Figure 3A,A′). When normalized to body weight, SOL muscle showed reduction (−10%, *p* < 0.05 for females, −12%, *p* < 0.05 for males) (Figure 3B,B′). Relative soleus mass showed a significant age × genotype interaction (*p* < 0.0001), indicating that the genotype effect became more pronounced with age after normalization to body weight. At 5 weeks, relative soleus mass was similar between groups, whereas at 15 weeks *Winnie* mice showed reduced relative soleus mass. Relative SOL mass showed significant effects of sex (*p* < 0.05), age × genotype (*p* < 0.001), and genotype × sex (*p* < 0.05), indicating that the effect of genotype on muscle mass changed with age and differed by sex. Absolute TA muscle mass was decreased (−27%, *p* < 0.05 for females, −23%, *p* < 0.001 for males) (Figure 3C,C′). When normalized to body weight, TA muscle mass decreased (−9%, *p* < 0.01 for females; −13%, *p* < 0.01 for males) (Figure 3D,D′). Relative TA mass showed a significant age × genotype interaction (*p* < 0.0001), indicating age-dependent progression of the genotype effect. Relative TA mass was similar at 5 weeks but was reduced in *Winnie* mice at 15 weeks. Relative TA mass also showed a significant age × genotype × sex interaction (*p* < 0.001) and a significant genotype × sex interaction (*p* < 0.05), suggesting that the effect of genotype on TA mass depended on both age and sex. These results indicate that muscle wasting in *Winnie* mice was not uniform across sex or muscle type. The genotype × sex interactions suggest that males are more susceptible to disease-associated muscle loss, supporting a sex-dependent phenotype rather than a simple genotype effect.

### 3.4. Histological Evidence of Myofiber Atrophy of SOL and TA Muscles of Winnie Mice

**Figure 4:**
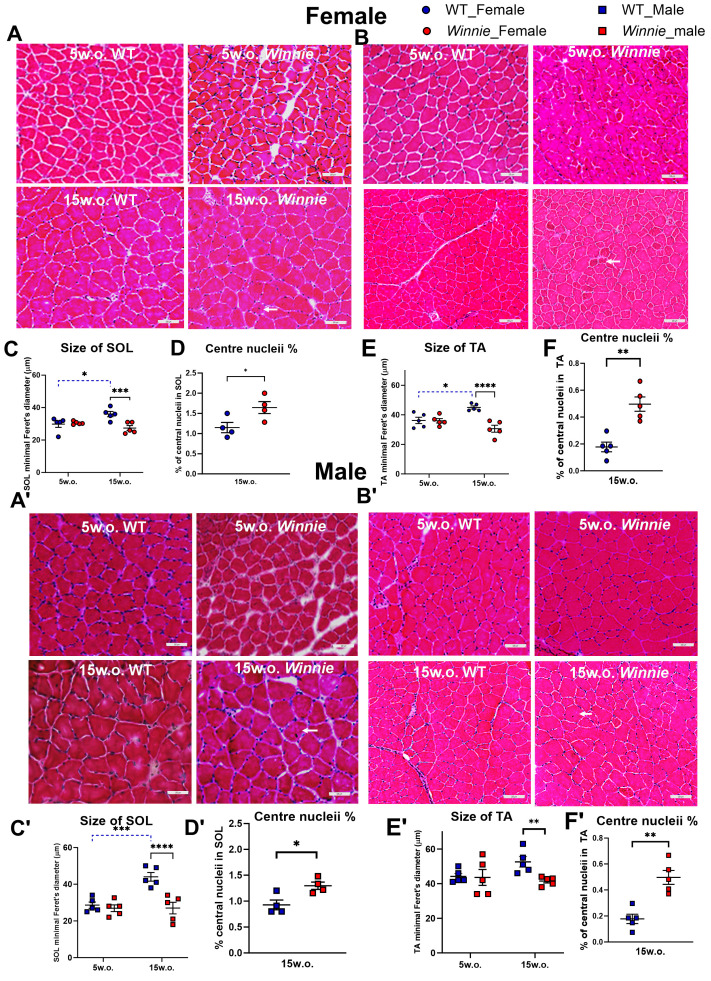
Histological evidence of myofiber atrophy and central nucleation in *Winnie* mice. H&E-stained cross-sections for SOL (**A**,**A′**) and TA (**B**,**B′**) muscles of female and male *Winnie* mice compared to WT mice at 5 weeks and 15 weeks. Scale bar, 100 µm. Minimal Feret’s diameter of SOL fibers of female and male *Winnie* mice compared to WT mice at 5 and 15 weeks (**C**,**C′**). Minimal Feret’s diameter of TA fibers of female and male *Winnie* mice compared to WT mice at 5 and 15 weeks (**E**,**E′**). % of central nuclei in SOL fiber in *Winnie* mice compared to WT mice at 15 weeks (**D**,**D′**). % of central nuclei in TA fiber in *Winnie* mice compared to WT mice at 15 weeks (**F**,**F′**). Data are mean ± SEM. Each symbol represents one mouse. Arrow indicates centrally nucleated fiber. Each symbol represents one mouse; fiber diameter analyses were performed in *n* = 5 mice/group, and central nucleation was assessed in 15-week mice only (soleus *n* = 4 mice/group; tibialis anterior *n* = 5 mice/group). * *p* < 0.05, ** *p* < 0.01, *** *p* < 0.001, **** *p* < 0.0001.

To determine whether chronic colitis is associated with structural myofiber pathology, we examined H&E-stained cross-sections of SOL and TA muscles in female and male *Winnie* and WT mice at 5 and 15 weeks of age. At 5 weeks, fiber morphology and size appeared comparable between *Winnie* and WT mice. By contrast, 15-week-old *Winnie* mice showed smaller fibers in both SOL and TA compared with age- and sex-matched WT controls (Figure 4A,B,A′,B′). Quantitative analysis confirmed a significant reduction in minimal Feret’s diameter in SOL fibers of 15-week-old *Winnie* mice (females: −23%, *p* < 0.01; males: −38%, *p* < 0.01) and in TA fibers (females: −32%, *p* < 0.001; males: −21%, *p* < 0.05), with no differences at 5 weeks (Figure 4C,C′,E,E′). Histological analysis showed muscle fiber atrophy at 15 weeks, with reduced fiber diameters in both SOL and TA muscles. SOL fiber diameter showed a significant age × genotype interaction (*p* < 0.0001), indicating that the effect of genotype on fiber size increased with age. Fiber diameters were broadly similar at 5 weeks, but *Winnie* mice showed smaller soleus fibers at 15 weeks. SOL fiber diameter also showed a significant genotype × sex interaction (*p* < 0.0001), indicating that the effect of genotype on soleus morphology differed by sex. TA fiber diameter also showed a significant age × genotype interaction (*p* < 0.0001), indicating that the genotype effect on fiber size became stronger with age. At 5 weeks, TA fiber diameter was similar between groups, whereas at 15 weeks *Winnie* mice showed reduced TA fiber diameter. TA fiber diameter showed significant genotype × sex (*p* < 0.01), and age × genotype × sex effects (*p* < 0.01), indicating a complex interaction among disease progression, sex, and muscle morphology. The larger genotype-associated reduction in soleus fiber diameter in males than in females suggests greater male susceptibility to *Winnie*-associated fiber atrophy. The proportion of fibers containing central nuclei was also increased in 15-week-old *Winnie* mice in both SOL and TA for females and males (*p* < 0.05–0.01) (Figure 4D,F,D′,F′). Though levels remained low (~2–5%), consistent with mild chronic remodeling, the increase in central nucleation suggests ongoing muscle damage-repair processes.

Overall, the consistent age × genotype interaction across outcomes indicates that genotype-related differences became progressively more evident with age. At 5 weeks, *Winnie* and WT mice were largely similar, whereas by 15 weeks *Winnie* mice showed clear deterioration in inflammatory, physical activity, and structural measures.

### 3.5. Fiber Size Distribution of SOL and TA Muscles of Winnie Mice

**Figure 5:**
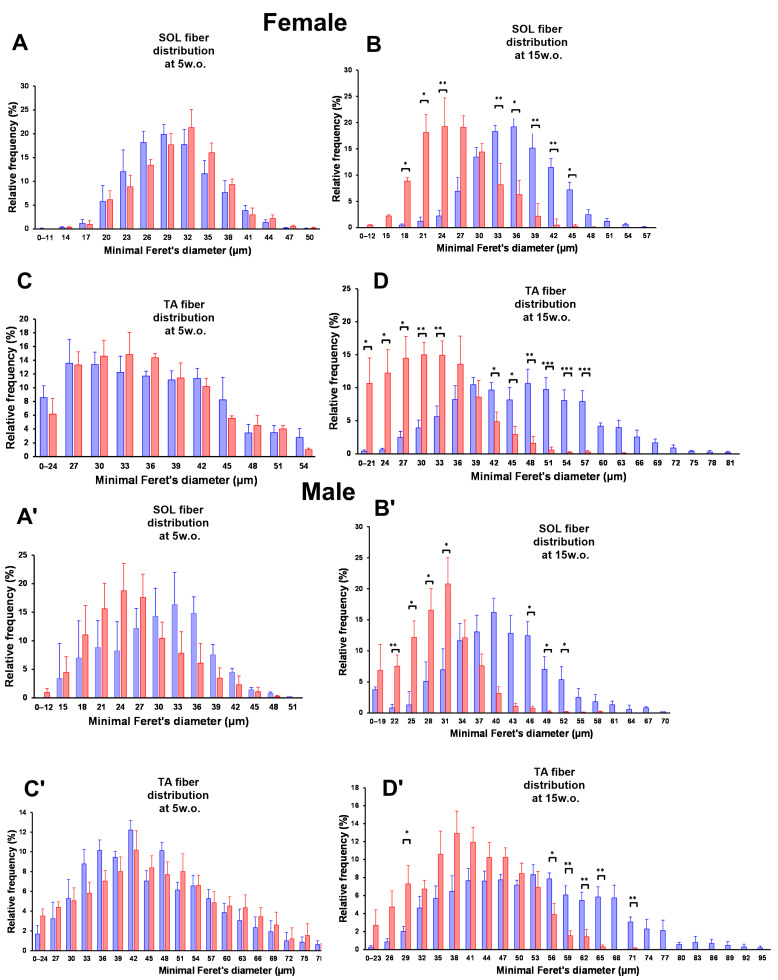
Fiber size distributions in SOL and TA of *Winnie* and WT mice. SOL fiber size distribution of female and male *Winnie* mice compared to WT mice at 5 weeks (**A**,**A′**). SOL fiber size distribution of female and male *Winnie* mice compared to WT mice at 15 weeks (**B**,**B′**). TA fiber size frequency distribution of female and male *Winnie* mice compared to WT mice at 5 weeks (**C**,**C′**). TA fiber size distribution of female and male *Winnie* mice compared to WT mice at 15 weeks (**D**,**D′**). Overlaid lines depict the distribution trend for each genotype. Red bars indicate WT mice and blue bars indicate *Winnie* mice. Data are mean ± SEM; *n* = 5 mice/group; * *p* <0.05, ** *p* < 0.01, *** *p* < 0.001.

At 5 weeks of age, SOL and TA fiber size distributions were broadly comparable between WT and *Winnie* in both females and males, with largely overlapping histograms across most diameter bins. By 15 weeks, clear genotype-dependent shifts in fiber size distributions emerged. In female SOL, *Winnie* showed a left-shift toward smaller minimal Feret’s diameters with reduced representation of larger fibers compared with WT, with multiple bins significantly different between genotypes. Female TA at 15 weeks similarly demonstrated an enrichment of smaller fibers in *Winnie* and a relative reduction in larger-diameter fibers versus WT across several bins. In males at 15 weeks, SOL and TA distributions also shifted toward smaller diameters in *Winnie* compared with WT, again with multiple significant bin-wise differences and a visibly diminished right-hand tail (larger fibers) in *Winnie* (Figure 5A,A’,D,D’). Overall, these patterns indicate that differences between WT and *Winnie* are minor at 5 weeks but become pronounced by 15 weeks in both muscles and both sexes.

### 3.6. Association Between Intestinal Inflammation and Muscle Atrophy of Winnie Mice

**Figure 6:**
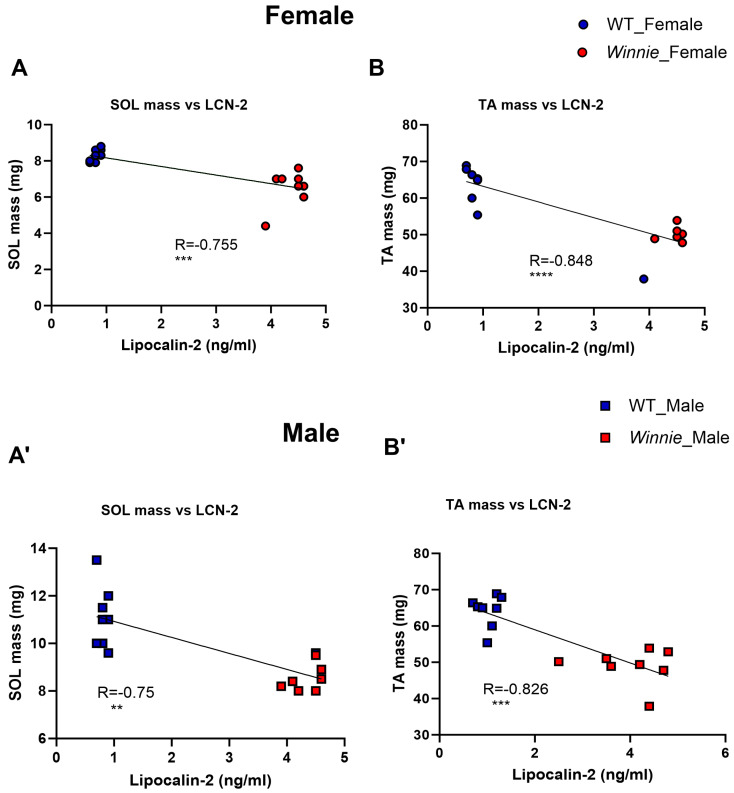
Correlation between fecal lipocalin-2 (LCN-2) and muscle mass in female and male *Winnie* mice. Scatter plots with regression lines (R values shown) depict relationships between fecal LCN-2 (ng/mL) versus SOL/TA mass (mg) of *Winnie* mice compared to WT mice. LCN-2 levels versus SOL mass of *Winnie* mice compared to WT mice (**A**,**A′**). LCN-2 levels versus TA mass of *Winnie* mice compared to WT mice (**B**,**B′**). Each symbol represents one mouse. Solid lines indicate linear regression fits. Correlation analyses were performed using paired data from mice with available measurements for both fecal LCN-2 and muscle mass. R: Spearman coefficient. *n* = 16 mice/group. ** *p* < 0.01, *** *p* < 0.001, **** *p* < 0.0001.

LCN-2 levels strongly negatively correlated with muscle mass at 15 weeks. SOL mass correlated negatively with fecal lipocalin-2 in females (R = −0.755, *p* < 0.001) and males (R = −0.750, *p* < 0.01) (Figure 6A,A′). TA mass also correlated negatively with fecal lipocalin-2 in females (R = −0.848, *p* < 0.0001) and males (R = −0.826, *** *p* < 0.001) (Figure 6B,B′).

### 3.7. Association Between LCN-2 and Wheel Activity of Winnie Mice

Fecal LCN-2 negatively correlated with wheel running distance (R = −0.71, *p* < 0.01 for both females and males) and velocity (female R = −0.71, *p* < 0.01; male R = −0.88, *p* < 0.0001), linking higher LCN-2 to impaired performance across sexes (Supplementary Figure S1).

## 4. Discussion

Muscle wasting contributes substantially to IBD-related disability [30], but its association with colitis severity across disease stages remains incompletely defined. In this study, age-stratified cohorts of *Winnie* mice showed functional differences at 5 weeks and more pronounced inflammatory, behavioral, and structural abnormalities at 15 weeks, consistent with stage-related worsening across separate cohorts. These findings support the *Winnie* mouse as a clinically relevant preclinical model for investigating IBD-associated muscle wasting and the gut–muscle axis.

Fecal LCN-2 tracked colitis severity and served as a sensitive marker of intestinal inflammation in this model [31]. The age × genotype interactions observed across outcomes indicate that phenotype differences became more pronounced in the older cohort, while the lack of strong sex effects for LCN-2 suggests that this biomarker primarily reflects disease stage rather than biological sex.

Voluntary wheel-running behavior was altered in *Winnie* mice, with subtle differences already detectable in the 5-week cohort and more pronounced impairments in the 15-week cohort. Early daytime reduction in wheel time in females should be viewed as an early activity change rather than proof of intrinsic muscle dysfunction.

At 15 weeks, *Winnie* mice showed reduced wheel-running distance and time, lower velocity, and increased inactivity, concurrent with more marked colitis. In the 5-week cohort, wheel-running behavior was largely driven by circadian phase, with only limited genotype-related differences, suggesting that early activity changes represent a subtle functional signal. In the 15-week cohort, wheel-running distance showed a significant day-night × genotype × sex interaction, with a larger genotype-associated reduction in males than in females. The absence of a sex effect for wheel velocity and wheel time, together with significant time × genotype effects, suggests that some locomotor endpoints are more sensitive to circadian modulation than to sex alone. Overall, the wheel-running outcomes reflect a combination of inflammatory burden, sex, and daily activity phase rather than a simple genotype effect. Because these were separate age cohorts, the findings are best interpreted as age-stratified activity differences consistent with greater impairment in the older cohort, rather than direct proof of within-animal temporal decline. Voluntary wheel running is a composite behavior influenced by motivation, sickness behavior, circadian phase, discomfort, and general well-being. Therefore, although, reduced wheel performance is consistent with altered physical activity, it does not by itself establish intrinsic skeletal muscle dysfunction.

Structural analyses showed reduced muscle mass and smaller fiber diameters in the 15-week cohort. The genotype × sex effects in TA mass and soleus fiber diameter show that structural muscle loss is sex-dependent rather than evenly distributed across cohorts. The stronger phenotype in males suggests greater male susceptibility to chronic colitis-driven atrophy [32]. The differences between TA and SOL suggest muscle-type-specific vulnerability, with fast/glycolytic muscle appearing more affected than slow/oxidative muscle, suggests inflammation-associated catabolism [33,34]. These findings are consistent with sex- and muscle-type-dependent susceptibility to inflammation-associated myopathy, and they support a gut–muscle link in chronic intestinal inflammation.

These structural changes correlated quantitatively with colitis biomarkers, as fecal LCN-2 showed strong negative correlations with muscle mass. The correlations between inflammatory marker, physical activity, and muscle mass suggest a direct link between intestinal inflammation and systemic muscle alterations, supporting gut–muscle crosstalk [35]. The consistent age × genotype interaction across inflammatory, functional, and muscle endpoints indicates that *Winnie*-associated pathology is more pronounced in the older cohort rather than remaining static across age-stratified comparisons. This pattern is consistent with limited changes in the 5-week cohort and more marked activity and skeletal muscle abnormalities in the 15-week cohort. Taken together, the biomarker and histological findings show that more pronounced intestinal inflammation is associated with sex- and muscle-type-dependent skeletal muscle deterioration, with the greatest abnormalities observed in the 15-week cohort.

Overall, this study provides the first comprehensive characterization of myopenia in a spontaneous IBD mouse model. It also shows that colitis severity, physical activity, and skeletal muscle alterations are linked, validating *Winnie* mice as a useful system for studying disease-associated muscle wasting. Study limitations include the absence of direct functional assays such as grip strength, in vivo force measurement, or ex vivo contractility, observational cross-sectional design, reliance on a single model, lack of therapeutic intervention testing, and the use of only two age cohorts. Nonetheless, the spontaneous pathology, littermate controls, and integrated phenotyping make this model a useful platform for future mechanistic and longitudinal work.

**Figure 7:**
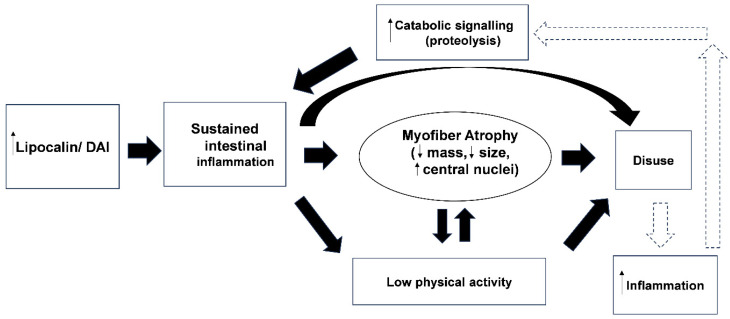
Proposed vicious cycle linking intestinal inflammation, myofiber atrophy, and reduced physical activity in *Winnie* mice. Higher levels of fecal LCN-2 and DAI reflect sustained intestinal inflammation, driving catabolic signaling and myofiber atrophy (low mass, fiber size, higher central nuclei). Atrophied muscle impairs voluntary wheel running, promoting disuse atrophy and perpetuating inflammation. The up/down arrows indicate increase and decrease, respectively, thick bold arrows indicate major proposed effects and dashed arrows denote hypothesized relationships derived from cross-sectional associations across age groups. Early interventions disrupting inflammation and catabolism can break this cycle.

These findings underscore the systemic nature of IBD and the importance of monitoring and maintaining muscle function. Figure 7 presents our proposed inflammation-atrophy-disuse cycle, integrating observed colitis-myopenia progression with hypothesized catabolic feedback loops amenable to early intervention. While evidenced by LCN-2 correlations and muscle atropy, mechanistic pathways (circulating cytokines, disuse amplification) require future validation.

## 5. Conclusions

This study identifies a myopenic phenotype and reduced voluntary activity in *Winnie* mice across age-stratified cohorts of chronic colitis. The findings are consistent with age-associated worsening of inflammatory, functional, and structural abnormalities, with the 15-week cohort showing more pronounced colitis and skeletal muscle alterations than the 5-week cohort. These data support the *Winnie* mouse as a relevant preclinical model for studying IBD-associated muscle wasting and for evaluating muscle-preserving interventions.

## Figures and Tables

**Table 1:** Cohort composition and endpoint-specific sample sizes across age, genotype, and sex groups.

Age	WT/Win	M/F	Mice Enrolled	DAI *n*	Wheel/Promethion *n*	LCN *n*	SOL Mass *n*	SOL Fiber Dia *n*	TA Mass *n*	TA Fiber Dia *n*	Reused Across Assays	Missing/Excluded
5 wks	WT	F	8	8	8	6	5	5	5	5	Yes	None
5 wks	WT	M	8	8	8	6	5	5	5	5	Yes	None
5 wks	Win	F	8	8	8	6	5	5	5	5	Yes	None
5 wks	Win	M	8	8	8	6	5	5	5	5	Yes	None
15 wks	WT	F	8	8	8	8	8	8	8	5	Yes	None
15 wks	WT	M	8	8	8	8	8	8	8	5	Yes	None
15 wks	Win	F	8	8	8	8	8	8	8	5	Yes	None
15 wks	Win	M	8	8	8	8	8	8	8	5	Yes	None

Footnote: n indicates the number of mice analyzed for each endpoint; differences in n reflect planned endpoint-specific analyses using subsets of the same animals. Abbreviations: M, male; F, female; WT, wild-type; Win, *Winnie*; DAI, disease activity index; LCN, lipocalin-2; SOL, soleus; TA, tibialis anterior; dia, diameter.

## Data Availability

The data that support the findings of this study are available from the corresponding author upon reasonable request.

## Supplementary Materials

The following supporting information can be downloaded at: https://www.mdpi.com/article/10.3390/muscles5020038/s1. Figure S1: Lipocalin-2 (LCN-2) versus wheel distance/velocity in *Winnie* mice (15 weeks). Scatter plots with linear regression lines (R values shown) depict relationships between fecal LCN-2 (ng/mL) and wheel running metrics. LCN-2 versus wheel distance in *Winnie* mice compared with WT mice (A,A’). LCN-2 versus wheel velocity in *Winnie* mice compared with WT mice (B,B’). Blue symbols indicate WT mice, and red symbols indicate *Winnie* mice. Each symbol represents one mouse. Solid lines indicate linear regression fits. Both running distance and wheel velocity were negatively correlated with fecal LCN-2 in females and males. Correlation analyses were performed using paired data from mice with measurements available for both fecal LCN-2 and wheel-running outcomes. *n* = 16 mice/group. *** *p* < 0.001, **** *p* < 0.0001.
