# Supplementary material for: Progressive Myopenia and Functional Decline in the Winnie Mouse Model of Chronic Colitis
Source: Muscles. 2026 May 12;5(2):38. doi: 10.3390/muscles5020038 (PMC13214686; doi:10.3390/muscles5020038)
Supplement: Supplementary file 1 [file muscles-05-00038-s001.zip › muscles-4203315-supplementary.pdf]

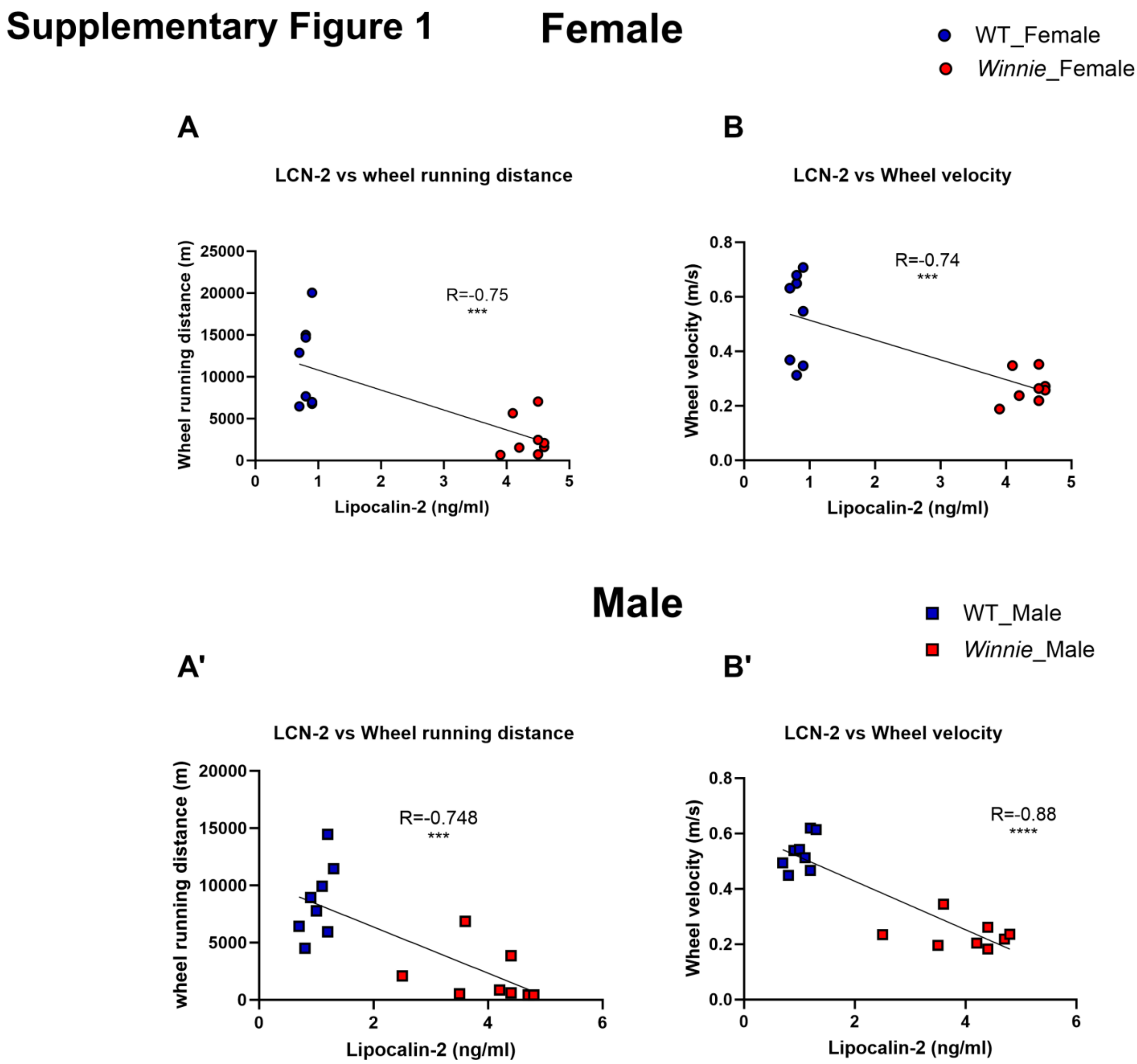

Figure S1. Lipocalin-2 (LCN-2) versus wheel distance/ velocity -in *Winnie* mice (15 weeks). Scatter plots with linear regression lines (R values shown) depict relationships between fecal LCN-2 (ng/ml) and wheel running metrics. LCN-2 versus wheel distance in *Winnie* mice compared with WT mice (**A, A′**). LCN-2 versus wheel velocity in *Winnie* mice compared with WT mice (**B, B′**). Blue symbols indicate WT mice, and red symbols indicate *Winnie* mice. Each symbol represents one mouse. Solid lines indicate linear regression fits.Both running distance and wheel velocity were negatively correlated with fecal LCN-2 in females and males. Correlation analyses were performed using paired data from mice with measurements available for both fecal LCN-2 and wheel-running outcomes. n = 16 mice/group. ***$p < 0.001$, ****$p < 0.0001$.
